# Impact of Medical Trainees on Efficiency and Productivity in the Emergency Department: Systematic Review and Narrative Synthesis

**DOI:** 10.5811/westjem.18574

**Published:** 2024-07-18

**Authors:** Jake Valentine, Jonathan Poulson, Jesus Tamayo, Amanda Valentine, Jacqueline Levesque, Shane Jenks

**Affiliations:** * University of Houston, Tilman J. Fertitta Family College of Medicine, Houston, Texas; † HCA Houston Healthcare, Kingwood, Kingwood, Texas; ‡ The University of North Texas Health Science Center, Fort Worth, Texas; § University of Texas Health Science Center at Houston, Houston, Texas

## Abstract

**Introduction:**

Effective medical education must balance clinical service demands for institutions and learning needs of trainees. The question of whether these are competing demands or can serve complementary roles has profound impacts on graduate medical education, ranging from funding decisions to the willingness of community-based hospitals and physicians to include learners at their clinical sites. Our objective in this article was to systematically review the evidence on the impact of medical trainees on productivity and efficiency in the emergency department (ED).

**Methods:**

We queried PubMed, Embase, Scopus, and Web of Science from earliest available dates to March 2023. We identified all studies evaluating the impact of medical students and/or residents in the ED on commonly used productivity and efficiency metrics. Only studies in EDs in the United States were included. No additional filters were used. We assessed the risk of bias of included studies using the Risk of Bias in Non-randomized Studies – of Interventions (ROBINS-I) tool. Certainty of evidence was rated using the GRADE (Grading of Recommendations Assessment, Development and Evaluation) approach. Study findings were combined in a narrative synthesis and reported according to PRISMA guidelines.

**Results:**

The literature search yielded 3,390 unique articles for abstract screening. Eighty-one abstracts were identified as relevant to our PICO question (population, intervention, control, and outcomes), 76 of which had retrievable full-text articles and the themes of which were discussed in a narrative synthesis. We selected 13 of the full-text articles for final inclusion in a systematic review. Studies were roughly split between observational (6) and quasi-experimental (7) designs. The majority of studies (11) were single-site studies. Only two studies could be graded as low risk of bias per the ROBINS-I tool.

**Conclusion:**

Low-GRADE evidence suggests that students and residents decrease ED efficiency by a statistically small effect size of debatable clinical importance. Residents provide a moderate boost to ED productivity. Students do not produce a statistically or clinically significant impact on ED productivity. Residents increase emergency department relative value units revenue by $26.30 an hour, while students have no impact. Both types of learners decrease efficiency.

Population Health Research Capsule What do we already know about this issue?

*Learners can be both an asset and a liability in terms of emergency department operations.*

What was the research question?

*What is the net impact of learners on efficiency and productivity in the emergency department.*

What was the major finding of the study?

*Residents increase emergency department relative value units revenue by $26.30 an hour, while students have no impact. Both types of learners decrease efficiency.*

How does this improve population health?

*This finding may guide stakeholders regarding decisions about reimbursement for education services or having learners in their department.*

## INTRODUCTION

There are conflicting opinions on the perceived value of medical trainees, stemming from their dual roles of learning and service. Trainees define value as maximizing learning opportunities and interactions with faculty. Attending physicians identify value as trainees completing clerical tasks and freeing up time for patient care. Administrators see value through the lens of addressing economic challenges of the hospital, with trainees providing potential value via documentation and improvement of organization-wide metrics—both of which are seen as low-value activities by trainees.^1^ It is not possible to sidestep the conflict between these roles, as time spent on teaching activities during an EM shift is independently associated with resident perceptions on the education value of the shift.^2^

There are several additional stakeholder considerations pertinent to our research question. Community preceptors are increasingly seeking compensation for teaching time.^3^ Current practices regarding compensation for teaching time of community preceptors are inconsistent.^3^ A logic model might posit that trainees decrease compensation to the supervising physician by decreasing relative value units (RVU) or increasing the amount of time spent post-shift via teaching, feedback, or deferring on-shift activities for these purposes. If this is the case, then this would strengthen arguments for compensating physicians for accommodating learners on shift. Of course, trainees also provide services that might be valued by attendings physicians, such as decreasing their documentation burden, arranging consults, and gathering patient histories.

Stakeholders in the administrator and hospital leadership category might be more or less inclined to enter an affiliation agreement with a medical school or graduate medical education (GME) program depending on the projected impact on important efficiency metrics. Lastly, and perhaps most controversially, there are high-stakes decisions regarding the continuation of indirect graduate medical education (IME) funding, which rely on the implicit assumptions that trainees increase the cost of care at least partly due to impacts on attending physician productivity and efficiency. This is based on data from training sites that historically have represented large, urban, university-affiliated hospitals, with limited generalizability to the majority of EDs across the country. Medicare has reimbursed hospitals for IME costs since 1983 based on the ratio of residents per hospital bed and the premise that the higher costs of patient care at teaching hospitals is due to the presence of trainees.^4^ In 2019, over $10 billion in IME payments were distributed to teaching hospitals, supporting roughly 90,000 residents at a cost of $110,000 per resident.^5^

Despite this clearly demonstrated need for data on the impact of trainees on efficiency and productivity in the ED, there are no randomized controlled trials, systematic reviews, or meta-analyses on the subject. In this article we aimed to fill that void by examining existing evidence on the topic, clarifying current gaps in the literature, and making suggestions for future research.

## METHODS

We queried PubMed, Embase, Scopus, and Web of Science from earliest available date to March 9, 2023. Search terms and search strategy were developed collaboratively by two content experts in medical education, one content expert in healthcare administration and public policy, and two research librarians. Our PICO question (population, intervention, control, and outcomes) was “What is the impact of learners in the (ED) on efficiency and productivity metrics?” The population for our question was learners. We included broader terms such as “trainee” and “learner” in our search strategy in case there was literature on non-traditional rotators such as students in undergraduate, scribe, or advanced practice practitioner programs. We also included synonyms used to describe medical students and resident such as “clerk” and “intern” (Appendix A).

The intervention of interest was presence of a learner, compared to absence of a learner. The learner had to be present in the ED and under the direct supervision of an emergency medicine attending. Our outcome was efficiency and productivity. Efficiency described how quickly patients moved through the department and included synonyms such as “throughput.” Productivity referred to how much work an attending physician was able to complete, most commonly measured by patients seen or RVUs generated per unit of time (Appendix A). These PICO characteristics were captured by the search terms outlined in Appendix B.

The first author reviewed the initial 6,175 results to ensure that the automatically detected duplicates were appropriate and manually excluded undetected duplicates. The remaining 3,390 abstracts were screened by two reviewers to judge whether the article would satisfy our PICO question, with differences adjudicated by a third reviewer. Reviewers worked independently and were blinded to the result of the first vote for cases in which they served as second reviewer. Adjudication also occurred blindly, without access to the individual reviewer’s votes. Of these 81 abstracts, 76 were available for full-text review. Full-text reviews were performed by the first author with notations describing justification for proposed inclusion or exclusion. Annotated full texts were then put to a consensus vote among the four reviewers. We applied an additional inclusion criterion of studies conducted in the United States during this step, owing to differences in training and supervision requirements for medical trainees in other countries. Records were compiled using Covidence systematic review software (Veritas Health Innovation, Melbourne, Australia).

The references sections of included studies were hand-checked for additional candidates for inclusion, but this search did not reveal any new studies to add to the final inclusion list. Data was extracted by the first author and organized by population and outcome. The impact of residents and students on the outcome categories were displayed separately when possible. We constructed tables and figures according to the *Cochrane Handbook for Systematic Reviews of Interventions*.^6^ A standardized effect size (Cohen *d*) with 95% confidence intervals was calculated for studies that provided the necessary data. We rated certainty of evidence using the Grading of Recommendation, Assessment, Development and Evaluation (GRADE) approach, adapted for reviews not including a single estimate of effect.^7^

We assessed risk of bias was assessed using the Risk of Bias in Non-randomized Studies – of Interventions (ROBINS-I) tool,^8^ with notations made through Covidence and consensus assessment voted upon by the four reviewers. Effect measures were presented as reported by each included article. We presented results of the systematic review and narrative synthesis according to PRISMA guidelines.^9^

## RESULTS

**Figure 1.**
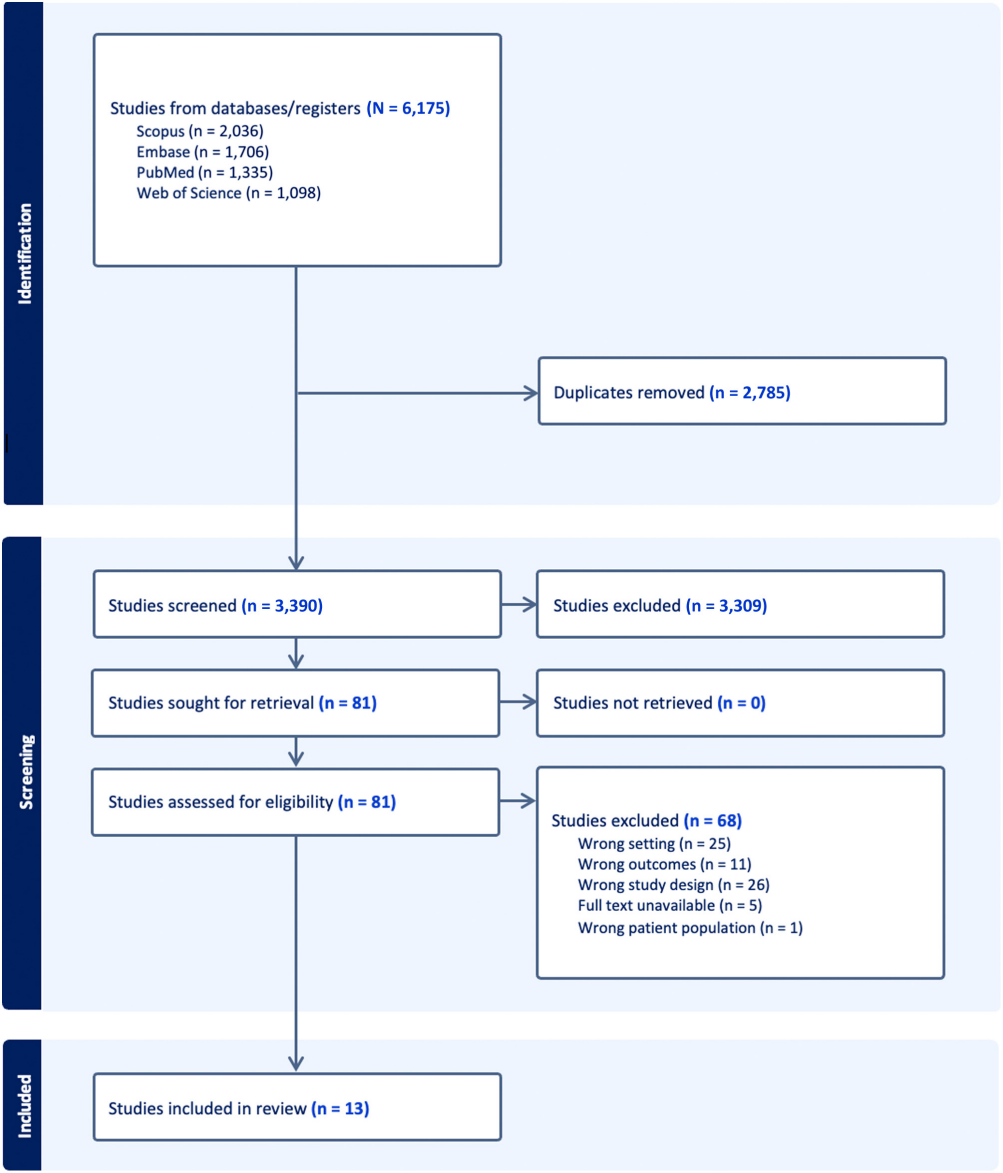
PRISMA* flow diagram. *PRISMA*, Preferred Reporting Items for Systematic reviews and Meta-Analyses.

A search of PubMed, Embase, Scopus, and Web of Science identified 6,175 articles with 2,785 duplicates, leaving 3,390 article abstracts to be screened. We excluded 3,309 abstracts that did not address our PICO questions, yielding 81 full-text articles sought for retrieval, five of which were unavailable as they were abstract-only publications. Themes from these 76 articles are discussed in the narrative synthesis, with a final 13 selected for inclusion for the systematic review (Figure 1). Characteristics of included studies are summarized in Appendix C.

### Themes of Excluded Studies

Common reasons for exclusion after full-text review were wrong setting or wrong outcome. Some studies evaluated fellows^10^ or moonlighting residents^11^ ^,^ ^12^ who were practicing independently and not being directly supervised by an EM attending. An Emory-based study showed EM-trained critical care fellows caring for boarding intensive care unit patients generated an additional 3.07 RVUs/hour for the department.^10^ Northwestern University evaluated the economics of paying residents to serve as triage physicians in a moonlighting role in their ED and found that the return on investment from “left without being seen” charge capture was +54%.^11^ Svirsky et al (2013) showed a similar moonlighting program reduced length of stay (LOS) by 25 minutes. These studies were not included in our review as the residents and fellows were neither acting in a learner role nor being supervised by the on-shift EM attending physician.

Several excluded studies involved learners in the ED on non-EM services who were not supervised by the on-shift EM attending. Replacing a surgical resident with an MLP (midlevel practitioner) during protected education time added 67 minutes to ED LOS.^13^ Resident presence on a trauma rotation decreased ED LOS for admitted trauma patients.^14^ Residents on a trauma consult service did not take any longer than attending surgeons to complete consultations.^15^ Presence of an in-house cardiology fellow decreased door-to-balloon times for ST-segment elevation myocardial infarction (STEMI).^16^

Some studies evaluated the impact of learners on efficiency or throughput measures but compared them to MLPs or simultaneously adjusted attending physician staffing.^16^ ^–^ ^18^ McGarry et al (2010) used a pre-post design evaluating LOS after a new EM residency program was created at their hospital. They redistributed 33% of the attending physician coverage toward the low-acuity “urgent-care” area of their department. There was a slight increase in LOS post-implementation of the residency program, but greater differences may have been masked by the fact that the low-acuity area—the area most likely to be bottlenecked by clinician efficiency—had more coverage. French et al (2002) found that patients waited an additional 20 minutes for disposition decisions when residents were absent on conference days. However, conclusions regarding learner presence would be difficult to make, as they replaced the roughly 60 hours of resident coverage with 40 hours of MLP and faculty coverage. Clearly, residents were viewed as assets requiring increased staffing to offset their absence, but these studies compare trainee performance to MLP or attending physician performance rather than strictly presence vs absence of learners and were thus omitted from our review.

Other studies took place in a pediatric-only ED. These studies generally demonstrated an increase in LOS with resident and/or medical student teams, likely mediated through increased laboratory and imaging utilization.^19^ ^,^ ^20^ ^,^ ^21^ Jadhav et al (2019) showed a clear association between resident involvement in a case, the number of studies ordered, and the LOS increase for those cases. Corey et al (2022) redemonstrated these findings and also showed that resident involvement was linked with an increase in RVUs/patient, again likely mediated by increased test utilization. While this result is potentially positive from a hospital administration perspective in that it increases revenue, it also likely represents low-value care that inflates the patient’s bill and the cost of healthcare as a whole. We did not include studies from pediatric EDs as they have very different operational characteristics than adult or combined EDs.

There were multiple studies in other countries using a natural experimental condition offered by junior physicians going on strike, but the applicability of these junior doctors to resident physicians in the US is limited due to differences in training and supervision requirements.^22^ ^,^ ^23^ ^,^ ^24^ ^,^ ^25^ Studies from Korea,^22^ New Zealand,^23^ Spain,^24^ and Australia^25^ all provide interesting case studies, but the “junior doctor” terminology is variably defined, sometimes referring to independently practicing physicians and at other times referring to physicians in training.

Studies excluded on the basis of wrong outcome tended to measure patient satisfaction or quality of care metrics. Perhaps most interestingly, Michael et al (2022) showed that EM residents improved time-limited quality metrics for stroke, sepsis, and STEMI, while off-service residents in the ED had a negative impact.^26^

### Narrative Synthesis of Included Studies

The 13 included studies addressed our PICO question by isolating presence vs absence of learner as the intervention and comparison groups, restricting the population to learners in the ED under the supervision of the on-shift emergency medicine attending physician, and included a measure of efficiency or productivity.

**Figure 2.**
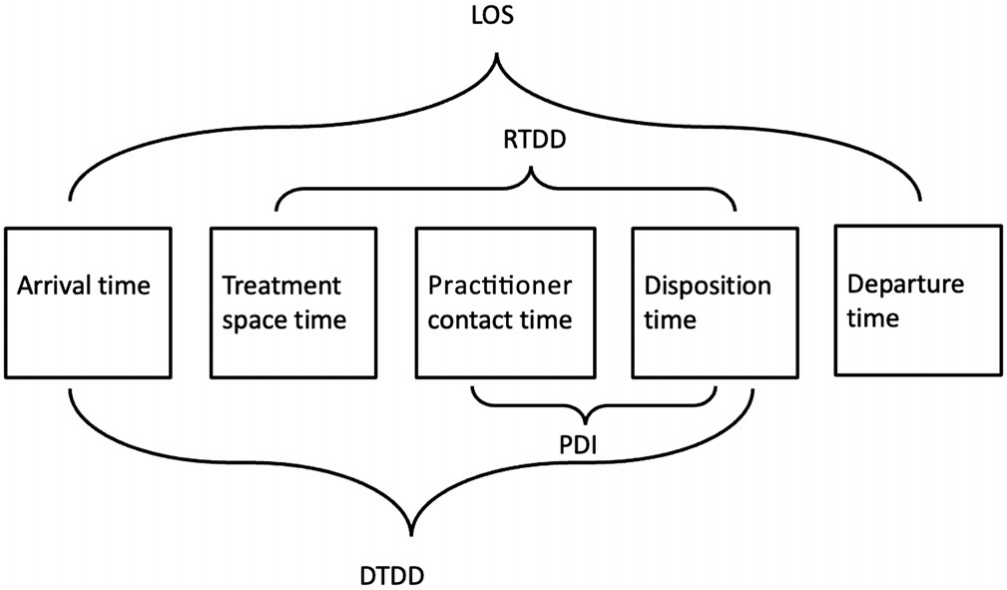
Emergency Department Benchmarking Alliance time stamps and intervals. *LOS*, length of stay; *DTDD*, door to disposition decision; *RTDD*, room to disposition decision; *PDI*, practitioner disposition interval.

Efficiency metrics were as defined by the Emergency Department Benchmarking Alliance Consensus Summit (Figure 2).^27^ Non-standardized definitions are explained in relation to the figure. Other abbreviations encountered included dLOS, which stands for length of stay for discharged patients, and TT, which is the interval from treatment space time “to when [the patient] is either discharged or admitted to the hospital.”^28^ It was unclear whether the endpoint of this interval was disposition or departure time, and the authors did not respond to email inquiries requesting clarification.

Seven studies assessed the impact of residents on efficiency in the ED (Table 1). Most (6/7) of the studies showed a small decrease in efficiency. The measured impacts on efficiency were all statistically small (Cohen *d* ranged from −0.15 to 0.15), but the clinical significance of the impact is more difficult to determine. The net difference in efficiency ranged from (−58) to 73 more minutes spent in the ED, with studies clustering reasonably well around the median value of 26 minutes. At academic departments with very long average LOS, 26 minutes may not be a meaningful increase. These seven studies, with the exception of the Pitts et al study (2014), were conducted at a single academic site.^32^ Each study, with the exception of Lammers et al (2003),^31^ included an average time metric above three hours, helping to explain the small statistical effect size. We identified three issues with interpretation of clinical meaningfulness of the impact of resident presence on ED efficiency.

**Table 1.** Study characteristics grouped by participant and outcome categories.

Study (measure)	Intervention N	Control N	Net difference (I–C)	Effect size (*d*)	95% CI [Lower, Upper]	Test	*P*-value
Efficiency – Residents							
Anderson et al, 2013^28^ (TT)	246 visits	7,689 visits	−58 minutes^t^	−0.15	[−0.27, −0.02]	K–S	0.02
DeLaney et al, 2013^29^ (LOS)	153,703 visits	40,331 visits	26 minutes^*^	N/A	N/A	K–W	<0.001
DeLaney et al, 2013^29^ (DTDD)	153,703 visits	40,331 visits	30 minutes^*^	N/A	N/A	K–W	<0.001
Kraut et al, 2020^30^ (RTDD)	4,537 visits	3,421 visits	10 minutes	0.10	[0.06, 0.15]	N/A	0.01
Lammers et al, 2003^31^ (DTDD)	N/A	N/A	40 minutes	N/A	N/A	*t*	<0.001
Pitts et al, 2014^32^ (LOS)	3,374 visits	25,808 visits	73 minutes^*^	N/A	N/A	N/A	<0.05
Robinson et al, 2020^33^ (PDI)	103,871 visits	7,283 visits	18 minutes	0.15	[0.13, 0.17]	*t*	<0.001
Efficiency – Students							
Chan and Kass, 1999^34^ (dLOS)	1,336 visits	639 visits	−5.4 minutes	−0.06	[−0.15, 0.04]	*t*	0.40
DeLaney et al, 2013^29^ (LOS)	13,949 visits	40,331 visits	24 minutes^*^	N/A	N/A	K–W	<0.001
DeLaney et al, 2013^29^ (DTDD)	13,949 visits	40,331 visits	15 minutes^*^	N/A	N/A	K–W	<0.001
Ioannides et al, 2015^35^ (LOS)	1,029,165 visits	343,696 visits	5.9 minutes	0.02	[0.02, 0.03]	*t*	<0.001
Smalley et al, 2014^36^ (dLOS)	6,880 visits	2,188 visits	14 minutes^*^	N/A	N/A	W	N/A
Efficiency – Mixed Trainees							
Dehon et al, 2015^37^ (LOS)	377 days	18 days	2.4 minutes	0.07	[−0.41, 0.54]	*t*	0.28
Productivity – Residents							
Bhat et al, 2014^38^ (PPH)	1,935 shifts	2,199 shifts	0.12 PPH	0.34	[0.28, 0.41]	*t*	<0.005
Clinkscales et al, 2016^39^ (RVUs/patient)	12,494 visits	11,560 visits	0.2 RVUs/patient	0.53	[0.50, 0.55]	*t*	<0.001
Robinson et al, 2020^33^ (PPH)	103,871 visits	7,283 visits	0.4 PPH	0.21	[0.19, 0.24]	*t*	<0.001
Productivity – Students							
Bhat et al, 2014^38^ (PPH)	514 shifts	2,199 shifts	0.0 PPH	0.00	[-0.10, 0.10]	*t*	0.99
Hiller et al, 2014^40^ (RVUs/shift)	101 shifts	101 shifts	0.13 RVUs/shift	0.01	[−0.27, 0.28]	*t*	0.95

t Denotes differences of medians. Mean values not explicit but were displayed graphically and acceptably close to stated median values.

* Denotes differences of medians.

*TT*, treatment time; *LOS*, length of stay; *DTDD*, door to disposition time; *RTDD*, room to disposition time; *dLOS*, discharge length of stay; *PDI*, practitioner disposition interval; *PPH*, patients per hour; *RVU*, relative value unit;

*t*, two-tailed Student *t*-test; *K–S*, Kolmogorov–Smirnov test; *K–W*, Kruskal-Wallis test; *W*, Wilcoxon rank-sum test.

Firstly, the varying measures of efficiency make synthesis of effect size problematic. Metrics that include the interval between disposition and departure time are influenced by boarding, which is a throughput bottleneck that is largely unaffected by resident presence in the ED and serves to attenuate any differences in efficiency between groups.^28^ ^,^ ^29^ ^,^ ^32^ The efficiency of the emergency team caring for a patient who spends several hours awaiting an inpatient bed is poorly reflected in these metrics.

Secondly, some of the studies excluded “fast-track” patients.^28^ ^,^ ^33^ Emergency departments have several names for these split-flow models, which all emphasize identifying patients with lower departmental resource needs with the aim of expediting their workup and disposition. These are the patient encounters in which efficiency is potentially most subject to the presence of a trainee, as there may be no competing throughput bottlenecks like imaging or laboratory turnaround time. Exclusion of fast-track patients attenuates measured efficiency differences.

Thirdly, selection and group allocation biases were present in varying degrees of the included studies. When the unit of analysis is the individual patient encounter,^30^ ^,^ ^32^ there is a high risk of selection bias as the residents may be preferentially opting for complex cases, which are more likely to meaningfully augment their learning. The presence of a resident is thus best viewed as a confounder, tightly associated with case complexity, which is a known driver of LOS.^41^ This limitation is perhaps most strongly present in the Pitts et al (2014) study.^32^ This nationally representative sample assessed LOS for patient encounters that included a resident compared to encounters with only an attending physician. While the authors attempted to limit confounding by adjusting for patient factors such as age and triage acuity, the nuances of patient complexity are likely to escape the simplification of a control variable. The authors’ suggestion that “residents see virtually all patients in major teaching EDs” appears empirically untrue given the multiple studies in our review that note the absence of learner involvement for lower acuity patients in split-flow ED models.

Group allocation bias arises when the propensity for attending physicians to work with residents is linked to differences in efficiency metrics. For example, physicians in education leadership (core faculty, assistant program directors, and program directors) would likely be pulled to attend conference. These physicians as a group may exhibit different practice patterns and efficiency trends than their colleagues.^42^ The direction and magnitude of this bias is difficult to guess owing to the paucity of literature on the topic. Robinson et al (2020) did a commendable job at controlling this bias by only including data from attendings with “balanced schedules”—meaning those who routinely work shifts both with and without residents.^33^ The rest of the studies that used shifts as the unit of analysis do not mention or control for this possible interaction between learner and attending schedules.^28^ ^,^ ^29^ ^,^ ^31^

Four studies assessed the impact of medical students on efficiency metrics in the ED. Most (4/5) showed that student presence had a statistically small decrease on ED efficiency, with impacts ranging from (−5) to 24 minutes and a median effect of 14 minutes. Similar difficulties as mentioned above precluded conclusions on the clinical significance of this impact. The efficiency measures generally included boarding time,^29^ ^,^ ^34^ ^–^ ^36^ although exclusion of fast-track patients was less of a threat for this group, as Chan and Kass (1999) and Smalley et al (2014) focused only on discharged patients.^34^ ^,^ ^36^ Selection bias was likely present in the Smalley study, since the medical students were part of a specially designated teaching service that only saw patients in particular treatment rooms, presumably in the higher-acuity areas of the ED.^36^

Group allocation bias was relatively well-protected against in the Ioannides et al (2015) study by using a natural experimental block design with students being pulled from the department to attend anesthesia training for the last week of each month-long rotation.^35^ Smalley et al used a similar condition of student absence during the last Friday of each rotation, during which students took their end-of-rotation exam.^36^ There would be a less obvious connection between attending characteristics (ie, involvement in education leadership) and learner presence under these conditions than with one based on resident conference time as commonly seen in the studies on resident impact of ED efficiency.

All three studies that measured resident impact on productivity showed a moderate to large statistical increase, with Cohen *d* ranging from 0.21–0.53.^33^ ^,^ ^38^ ^,^ ^39^ The clinical significance can perhaps be best illustrated in the following example, which assumes the average of level 4 charting, reimbursed at the 2022 rate of 2.74 RVUs per level 4 patient. The PPH difference (0.26) and RVUs/patient difference (0.2) add 0.76 RVUs/hour to an attending physician’s productivity.^43^ The Centers for Medicare and Medicaid Services reimbursed $34.61/RVU in 2022.^43^ Thus, a resident would bring an additional $26.30/hour of revenue from work RVUs to the ED. The two studies on medical student impact on productivity showed no statistically significant difference.^38^ ^,^ ^40^

Lastly, Dehon et al (2015) offered a unique analysis incorporating the total number of learners in the ED, inclusive of both students and residents.^37^ The correlation approach to total number of learners and efficiency and productivity metrics is interesting but potentially flawed in that there is reason to think resident and student presence has an interactive rather than cumulative effect. The suspicion that residents may mitigate student impacts on efficiency and productivity is reflected in Hiller et al’s (2014) observation that “residents performed the bulk of teaching and clinical supervision” of medical students.^40^

A summary of the certainty of evidence of included studies is provided in Table 2.

**Table 2.** Certainty of evidence of included studies.

Outcome	Effect	Number of studies	Certainty of the evidence (GRADE)
Resident impact on efficiency	Most (6/7) studies showed a small to moderate decrease in efficiency.	7	LOW ⊕⊕00 (due to serious risk of bias and indirectness)
Student impact on efficiency	Most (4/5) studies showed a small decrease in efficiency.	5	LOW ⊕⊕00 (due to serious risk of bias and indirectness)
Resident impact on productivity	All 3 studies showed an increase in productivity, centering around a medium effect size.	3	LOW ⊕⊕00 (due to serious risk of bias and moderate indirectness)
Student impact on productivity	Both studies showed no association with productivity.	2	LOW ⊕⊕00 (due to moderate risk of bias and severe indirectness)

*GRADE*, Grading of Recommendations Assessment, Development and Evaluation.

## DISCUSSION

Our systematic review evaluated 13 studies examining the effect of learner presence on efficiency and productivity in EDs. The majority of these studies (10 out of 13) showed moderate to severe risk of bias, leading to low-GRADE (Grading of Recommendations Assessment, Development and Evaluation) evidence for the four investigated outcomes. This bias was primarily due to potential confounders and indirectness of outcome measures, factors which led us to downgrade the review’s evidence level.

The low-GRADE evidence suggests that students and residents cause statistically small-to-moderate decreases in ED efficiency, with debatable clinical meaningfulness. Residents increase ED productivity by a statistically and clinically moderate effect size. Students do not produce a statistically or clinically significant impact on ED productivity.

We can review the implications of this review on the multiple parties involved in the decision to host medical students and residents at an emergency department.

Hospital and departmental administrators may continue to have concerns about learner impact on ED efficiency. This appears to be at most a modest effect and can be reframed as a necessary cost for ensuring a viable physician pipeline.

From a public policy standpoint, the role of trainees in the inefficiencies of teaching hospitals and increased cost of care should continue to be investigated. In EM, there is 24/7 bedside supervision, and most resource utilization and all disposition decisions are run by an attending physician. This may explain the non-intuitive suggestion that EM postgraduate year- (PGY) 3 residents slow down a department as much as interns do,^31^ possibly due to greater deference to the PGY-3 resident on medical decision-making, which increases laboratory and imaging utilization.^19^ ^–^ ^21^ Important differences likely exist between service lines in the relative contribution of trainees to inefficiency and decreased productivity. While cost-of-care differences were not directly addressed by this review, it would not appear that trainees in the ED are a large source of variance for efficiency or productivity. For attending physicians, this review should provide reassurance that having learners on shift will not negatively impact their RVU-based compensation.

Medical schools without salaried clinical faculty that have built-in teaching expectations will need to continue recruiting community preceptors. The decision to compensate these preceptors remains complicated.^3^ The review suggests that attending physicians balance teaching obligations without sacrificing productivity. However, this balance may be achieved by deferring less urgent clinical obligations, such as charting, until after shift end. This could create a significant, unmeasured time cost. Future studies might investigate the time spent after shifts when accommodating trainees, using time stamps pulled from electronic health records.

## LIMITATIONS

This review is subject to certain limitations. Inconsistencies in outcome reporting precluded meta-analysis. As an example, the relative contribution of a study with 1,935 shifts vs one with 103,871 patient encounters is difficult to reconcile, even if standardized effect sizes are available. Also, it would be challenging to combine an indirect efficiency measure like LOS with a more direct measure like door-to-disposition decision. The learner populations’ varying service lines and training levels added another layer of nuance precluding direct comparison between studies.

We identified four repeated themes leading to an increased risk of bias. First, the methods of group allocation were often unclear in the studies. A physician’s tendency to work shifts without trainees could relate to their efficiency or productivity. Only two studies controlled for this influence.^33^ ^,^ ^41^ Given the ethical issues that would arise from a randomized experimental design, having a natural experimental setting is likely the best evidence-generating opportunity that will be offered on the topic of trainees’ impact on ED efficiency and productivity. Future research should explicitly address or control this potential bias from group allocation.

Second, cross-sectional designs should continue to be used. Data from the Emergency Department Benchmarking Alliance have shown a consistent, gradual increase in ED LOS from 2010–2022,^44^ indicating a risk of maturation bias in pre-/post-residency designs.^31^ ^,^ ^39^ Third, LOS is not a reliable marker of ED efficiency as it is greatly influenced by external factors such as boarding times, which are beyond the ED’s control. Future studies should focus on metrics primarily influenced by ED operations.

Fourth, the unit of analysis should be shifts, not individual patient encounters. This approach can reduce the risk of reverse causation, where residents are preferentially assigned to more complex patients. The presence of this bias was highlighted in several studies.^29^ ^,^ ^30^ ^,^ ^32^ This would also control for varying practices among attending physicians in which some may independently see low-complexity cases that are felt to be unlikely to meaningfully contribute to learning.

The finding that residents may decrease ED efficiency and only modestly increase productivity may contrast with the experience of physicians at “resident-run” or “county” programs. Anecdotally, these are under-resourced departments that rely on residents to perform necessary services that would be offloaded to non-physician staff at other departments. These non-RVU-generating activities such as gathering equipment for procedures, starting intravenous lines, and transporting patients would not be captured in our study’s productivity metrics but are certainly necessary and value-added activities.

The legal mandate of EHR implementation as part of the 2009 American Recovery and Reinvestment Act may challenge applicability of earlier studies since part of resident and student efficiency benefits likely include offloading the documentation burden from the attending physician. However, the time increase spent charting and documenting post-EHR implementation seems to be small, not warranting the exclusion of pre-EHR studies.^45^

Historically, the vast majority of medical trainees have been located at university-associated hospitals that represent large, urban, tertiary-care, trauma centers. Thus, we expect any systematic review to be skewed toward these characteristics. Our review included 11 studies from academic sites and only one from a community site, which limits generalizability to EDs not matching those characteristics.

We recommend future research on this topic to focus on community training sites, given their increasing role in GME training.^46^ ^,^ ^47^ Applying findings from academic EDs to community EDs can be misleading due to differences in operational characteristics.^48^ The impact of trainees on efficiency and productivity could be more pronounced in smaller community EDs that serve a lower percentage of complex patients and do not employ the split-flow models commonly used in the studies included in our review.

## CONCLUSION

Our systematic review provides low-GRADE evidence that the presence of learners in the ED may modestly decrease efficiency. However, this effect may be offset by a similarly modest increase in attending physician productivity when supervising residents. Medical students do not impact attending physician productivity. The discussion highlights how these effects impact the multiple stakeholders in medical education and offers several considerations for future research on this topic.

## Supplementary Information



