# Supplementary material for: Impact of Medical Trainees on Efficiency and Productivity in the Emergency Department: Systematic Review and Narrative Synthesis
Source: West J Emerg Med. 2024 Jul 18;25(5):767–76. doi: 10.5811/westjem.18574 (PMC11418871; doi:10.5811/westjem.18574)
Supplement: Supplementary file 1 [file wjem-25-767-s001.docx]

Appendix A:

PubMed was queried via ‘MeSH+Title/Abstract’ search of keywords. For additional databases, MeSH terms were aggregated and entered manually, including truncation and wild card functions. Embase was searched using a ‘Title/Abstract’ filter of the above keywords. Scopus’ ‘Title/abstract/keywords’ filter was applied to our keyword search. Web of Science used a ‘Topic (title/abstract/keywords)’ filter into which we added our keywords. No additional filters or limits were used.

Concept 1: Learner

MeSH: "Students, Medical"[Mesh]

Keywords: “medical student*” OR resident* OR trainee* OR learner* OR clerk* OR intern* OR resident* OR fellow*

Concept 2: Emergency Department

MeSH: "Emergency Service, Hospital"[Mesh]

Keywords: “emergency department*” OR “emergency room*” OR “emergency ward*” OR “emergency unit*”

Concept 3: Throughput
 MeSH: "Length of Stay"[Mesh]

Keywords: throughput OR efficiency OR “length of stay” OR “left without being seen” OR “patients per hour” OR “patient per hour” OR “relative value unit*”

Appendix B:

Participant categories identified in our systematic review included; ‘residents’ (R), ‘emergency medicine residents’ (EM), ‘first-year emergency medicine residents’ (EM1), ‘second-year emergency medicine residents’ (EM2), ‘third-year emergency medicine residents’ (EM3), ‘off-service residents’ (OS), ‘nurse practitioner students’ (NP), ‘fourth-year medical students’ (MS4), ‘third-year medical students’ (MS3), and ‘sub-interns’ (SI). The intervention arm was presence of learners. The comparator arm was absence of learners. Outcome categories included ‘treatment time (TT)’, ‘length of stay (LOS)’, ‘discharge length of stay (dLOS)’, ‘door to disposition time (DTDD)’, ‘room to disposition time (RTDD)’, ‘provider to disposition interval (PDI)’, ‘patients per hour’ (PPH), ‘relative value units (RVUs) per hour’, and ‘RVUs per patient’. TT, LOS, dLOS, DTDD, RTDD, and PDI were subsequently grouped into the outcomes category of “efficiency”. PPH, RVUs/hour, and RVUs/patient were grouped into the outcome category of “productivity”.
